# Amylase quantification in the terminal Ileum following formation of an Ileostomy

**DOI:** 10.1038/s41598-020-76349-y

**Published:** 2020-11-09

**Authors:** D. A. Clark, T. Cuda, C. Pretorius, A. Edmundson, M. Solomon, A. D. Riddell

**Affiliations:** 1 Royal Brisbane and Women’s Hospital, Herston Road, Brisbane, QLD 4066 Australia; 2 Faculty of Medicine and Health, University of Sydney, and Surgical Outcomes Research Centre (SOuRCe), Missenden Road, Camperdown, Sydney, NSW 2050 Australia; 3 University of Qld, St Lucia, Brisbane, 4072 Australia; 4 St Vincent’s Private Hospital Northside, 627 Rode Road, Chermside, Brisbane, QLD 4032 Australia

**Keywords:** Biomarkers, Ileum

## Abstract

Amylase is elevated in the foregut and has been used to confirm anastomotic integrity after pancreatic surgery. The physiological activity of pancreatic enzymes in the ileum has been studied in healthy volunteers but not quantitated with the simple and readily available amylase measurements employed with serum tests. We aim to quantitate the levels of amylase in the terminal ileum. This was a prospective, non-randomised, non-blinded, consecutive cohort study conducted at the Royal Brisbane and Women’s Hospital. Consecutive patients undergoing routine surgery with an ileostomy were invited to participate in the study. Ileostomy effluent was collected and analysed daily for the first 5 post-operative days. This validation cohort included 8 males and 3 females, with a mean age of 49 years. Median daily amylase levels ranged from 4470 U/L to 23,000 U/L, with no specimens falling within the laboratory serum reference range of 40 to 130 U/L. Two specimens were not available on day one post-operative due to complete ileus. The sample size of 11 patients is small but was considered sufficient given that 55 effluent specimens were anticipated for analysis. Amylase levels remain highly elevated as the enzyme transits through the length of the small intestine and measured in the terminal ileum, and can be readily quantitated by the existing testing methodology routinely available.

## Introduction

Amylase is an enzyme predominantly produced by the pancreas and salivary glands, with the catalytic function of digestion of starch, glycogen and polysaccharides. There are two isoforms of secreted amylase; s-amylase secreted by the salivary glands and p-amylase secreted by the pancreas^1^. Insignificant levels are present in other organs. Most pathology labs measure total serum amylase including both isoenzymes. Serum amylase quantification is a standard pathology test available in almost all hospitals in the developed world. It is inexpensive^2^, simple and testing has a rapid turn around. The normal reference range varies with age, gender and between laboratories. Published reference ranges of amylase in the serum have been reported at 30–110 U/L^3^, or 20–300 U/L^2^ and 40–400 U/L in urine.

The functional activity of amylase in the gut has been studied by Holtmann et al.^4^. They found that the activity of pancreatic enzymes declined during aboral intestinal transit. The activity of enzymes in the small intestine was measured by placing an oro-ileal tube in healthy volunteers. The activity of amylase was shown to decrease by 43% ± 7% by the time it reaches the ileum, however absolute measures in U/L were not performed or reported.

Serum amylase levels are measured for the diagnosis of pancreatitis with a level of 2–4 times the upper limit of normal being considered diagnostic of acute pancreatitis. It is well recognised that serum amylase can be raised in a number of acute disease processes other than pancreatitis ^3,5^. Amylase levels in drain fluid can also be measured when there is suspicion of damage to the pancreas at surgery creating a pancreatic fistula. In 2005, The international study group on pancreatic fistula defined amylase levels of 3 times the serum levels on day 3 post operative, as diagnostic of a pancreatic fistula^6^. Other studies have used serum levels of greater than 1000 U/L on day one, post operative as a diagnostic criterion^3^.

Estimation of drain amylase has been employed in pancreatic surgery to detect anastomotic leaks at an early stage^7,8^. Lee et al. found a drain fluid amylase on post op day 1 of < 90 U/L demonstrated the highest negative predictive value of 98.2% for development of a pancreatic fistula^9^.

The aim of this study was to measure the levels of amylase present in the terminal ileum in the post-operative period following ileostomy formation. It was anticipated that the amylase levels in the terminal ileal effluent would be higher than the amylase levels expected in the systemic circulation. This information may be used to determine if fluid samples, collected from abdominal wounds or drains, are likely to reveal evidence of a communication with the ileal lumen. The ultimate application of this information may lay the foundation for the development of amylase as a biomarker of anastomotic leak in ileal pouch or surgery.

Despite an extensive literature search we were unable to find a published reference range of amylase levels quantified in the terminal Ileum. We describe the amylase levels in the terminal ileum in relation to normal serum levels.

## Methods

This was a prospective, non-randomised, non-blinded, consecutive cohort study. Consecutive patients, over the age of 18 years, who underwent an end or loop ileostomy in the colorectal surgical department of the Royal Brisbane and Women’s Hospital (RBWH) between January 2017 and April 2017 were included in the study. This constituted the selection criteria for inclusion in the study.

From days one to five, following ileostomy formation, a small sample of ileostomy effluent was collected daily, placed into a sterile tube and sent to the hospital pathology laboratory for standard amylase testing. All methods were carried out in accordance with relevant guidelines and regulations.

Demographic and clinical data together with ileal effluent amylase results were prospectively collected and entered into a database for analysis. Statistical analysis was performed with GraphPad Prism 8, GraphPad, San Diego, California, USA, and reviewed by a biostatistician. The median, interquartile range and the minimum and maximum amylase level for each day was reported (Table 1), since the data representing amylase levels did not fit a normal distribution.

**Table 1:** A summary of daily amylase levels in ileal effluent following ileostomy formation.

	Day 1	Day 2	Day 3	Day 4	Day 5
Number of values	9	11	11	11	11
Median (U/L) #	4470	16,400	23,000	20,000	11,000
25% Percentile	640	3070	9654	6640	7500
75% Percentile	21,469	53,500	45,190	53,800	16,200
Min (U/L)	190	1255	3340	1744	4170
Max (U/L)	74,200	37,000	93,500	106,600	38,000

^#^The normal amylase levels in serum is expected at 40–130 U/L, measured by the same pathology service.

This study was granted ethics approval by the Royal Brisbane & Women’s Hospital Human Research Ethics Committee, reference number: LNR/2018/QRBW/49591.

### Ethical approval and consent to participate

This study was approved by Royal Brisbane & Women’s Hospital Human Research Ethics Committee, reference number: LNR/2018/QRBW/49591. The research project meets the requirements of the National Health and Medical Research Council’s (NHMRC) National Statement on Ethical Conduct in Human Research (2007). Verbal consent was obtained from all patients and the waiver of written consent and breach of the Australian Privacy Principles were considered justified in accordance with National Statement 2.3.10 and approved. All data were de-identified before analysis.

## Results

This validation cohort included 8 males and 3 females, with a mean age of 49 years 4 months. The cohort was evenly distributed between oncology patients undergoing anterior resection for rectal cancer with a defunctioning ileostomy (n = 6) and patients undergoing resection (n = 5) for inflammatory bowel disease (IBD). As expected, the mean age in the IBD group was younger at 41 (+ /- SD 19) years of age, compared with the oncology group with a mean of 57 (± SD 15) years of age.

Whilst it was expected that an initial ileus or the peri-operative fast may lead to lower amylase levels on the first day post surgery, the median amylase level of 4470 U/L observed in our study was many times higher than the reference range for serum of 40 to 130 U/L (RBWH laboratory reference range). Two patients, however, did suffer an ileus on day 1 to the extent that there was no fluid effluent to test (Table 1).

**Figure 1:**
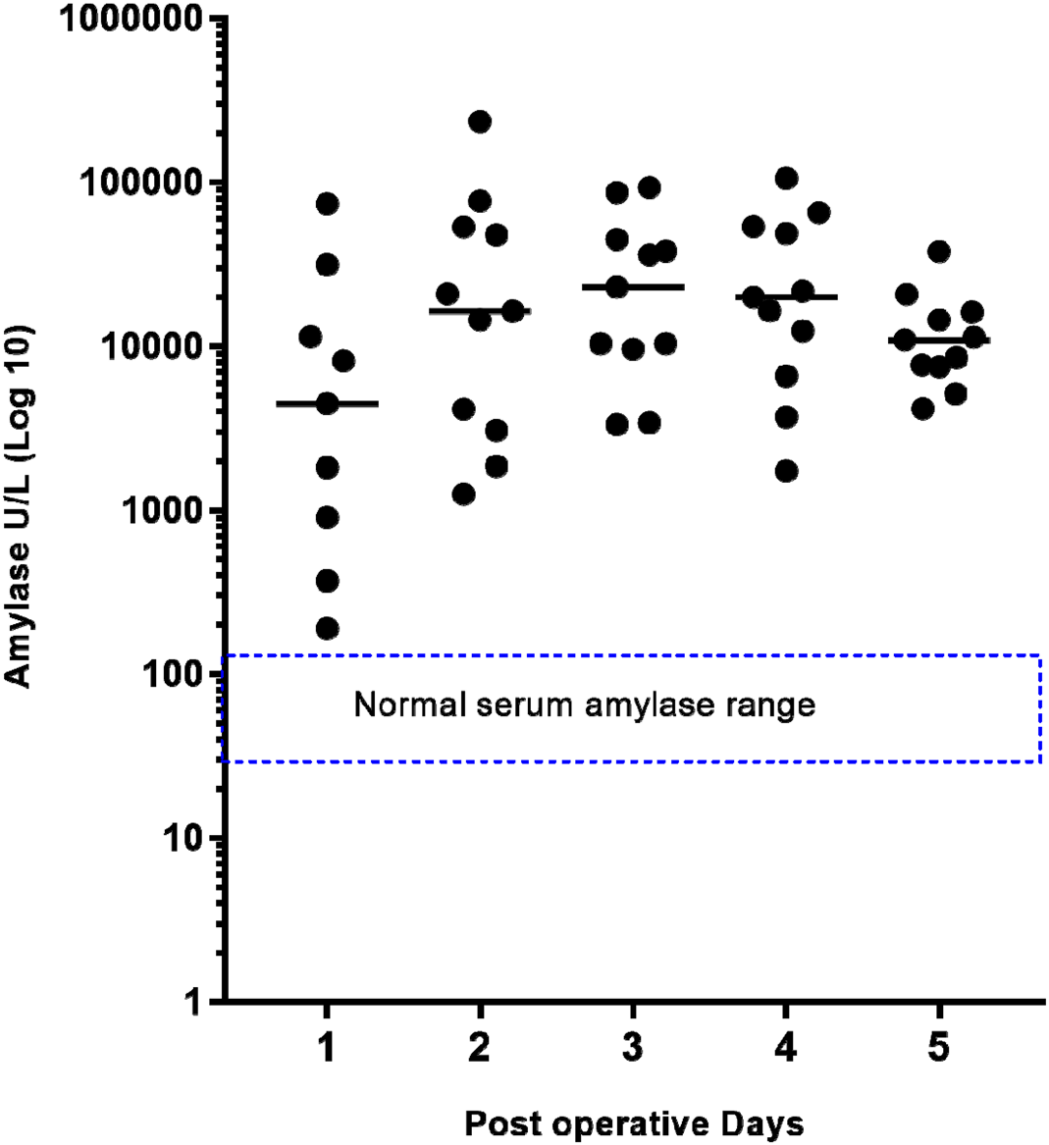
Daily amylase levels in ileal effluent post ileostomy formation represented with a logarithmic y axis and serum reference range overlaid. Bar marks the daily median amylase level.

We did not observe amylase levels in ileal effluent comparable to levels seen in normal serum. The measurements of amylase levels in ileal effluent were so elevated that a logarithmic scale was required to display them graphically (Fig. 1).

**Figure 2:**
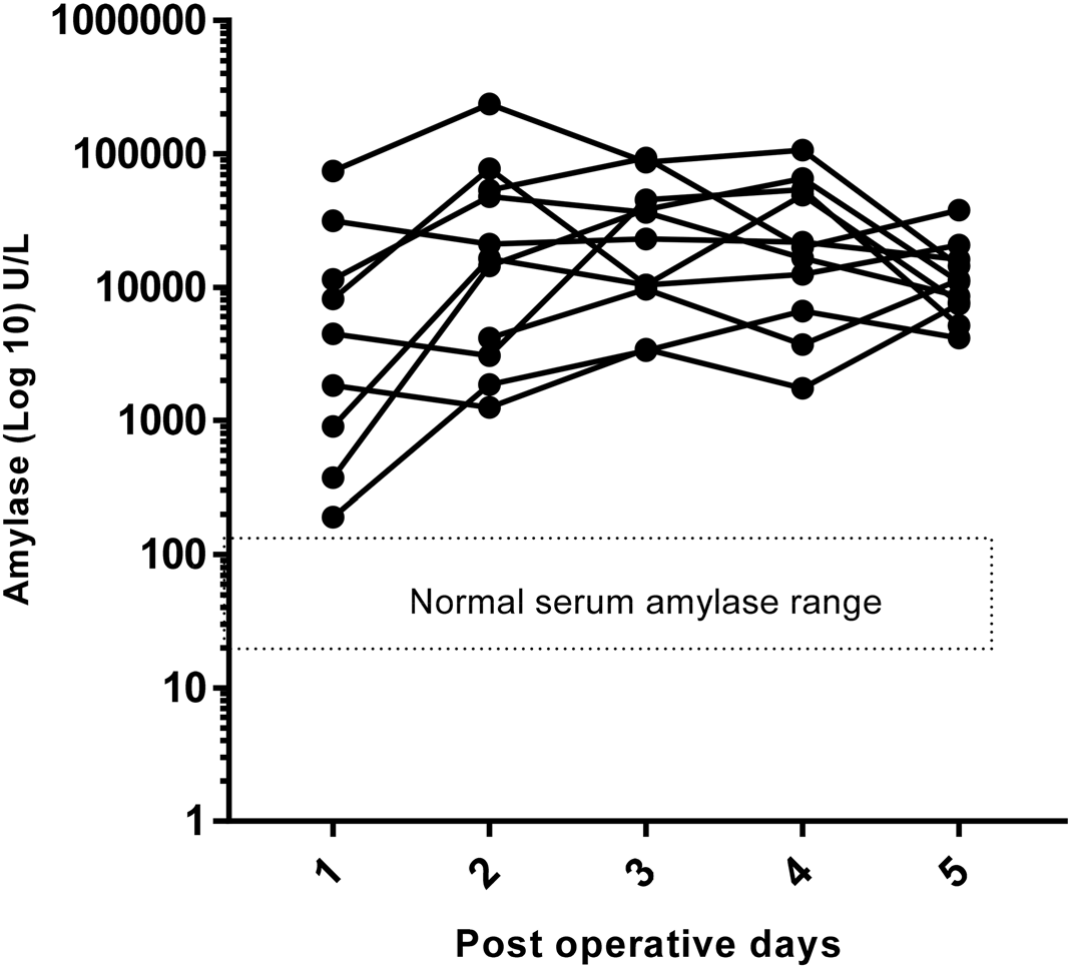
Daily trend of amylase levels, with serum reference range overlaid and represented with a logarithmic y axis.

Amylase levels remained high and steady in ileal effluent from day 2 to day 5 post ileostomy formation as demonstrated in Figs. 1 and 2.

## Discussion

The aim of this study was to provide evidence to indicate that levels of amylase in terminal Ileal effluent are higher than expected normal serum values and remain high after transit throughout the small intestine and in the early post-operative period. We observed amylase levels to be highly elevated in ileal effluent in the days (1 to 5) following ileostomy formation. Processing problems were surprisingly rare however three patients were lost to the study when a changeover of lab staff meant that samples were not processed over the weekend. Particulate matter present in the samples resulted in results coming back “haemolysed” on three occasions and further samples were collected.

At day one, (Day 1) of collection, two patients experienced a complete ileus resulting in no effluent available to sample for amylase measurement and consequently no results were recorded for the two patients on that day. It was relatively simple to prepare and process the samples in a standard manner as per the normal hospital collection protocols for amylase testing already established at the RBWH.

The enzyme amylase, which is produced in the salivary glands and pancreas, is in high concentrations in the foregut. We were able to establish that very high levels of this durable enzyme persist throughout the small intestine as measured in the terminal ileum. The difference, when compared to background or normal serum levels is striking. There is a more than 34-fold increase in the median amylase levels observed in terminal ileal effluent (4 470 U/L) compared to the higher limit of the reference range of normal in serum (130 U/L) on day one post-operative. The observed difference in levels was almost 175 times higher in the ileal effluent at day 3 (median 23,000 U/L) when compared to the upper limit of the normal serum reference range.

A weakness of this study is the small sample size of 11 patients but was considered sufficient given that 53 separate effluent specimens were obtained for analysis.

These findings are useful as fluid leakage from anywhere in the small intestine would be expected to return high levels of amylase. This knowledge could be applied to the sampling and analysis of abdominal wound or drain fluid to indicate a communication with the gut. In preliminary studies, the detection of amylase in pelvic drain fluid has been evaluated as a potential biomarker of anastomotic leak in ileal pouch surgery^10^. Sampling amylase in abdominal wound fluid may similarly be utilized to detect if there is evidence of a direct communication with the ileal lumen and may be an adjunct to the diagnosis of an enterocutaneous fistula.

## Conclusion

Amylase levels remain highly elevated as the enzyme transits through the length of the small intestine as measured in the terminal ileum. The measurement of amylase levels is inexpensive, readily available and results are rapidly returned.

## Data Availability

Supporting data is available on request.

## Abbreviations

RBWH: Royal Brisbane and Women’s Hospital
IBD: Inflammatory bowel disease
U/L: Units/Litre
